# The First Fifty Years of Finite-Time Thermodynamics

**DOI:** 10.3390/e28010049

**Published:** 2025-12-30

**Authors:** Bjarne Andresen, Peter Salamon

**Affiliations:** 1 Niels Bohr Institute, University of Copenhagen, Jagtvej 155 A, DK-2200 Copenhagen N, Denmark; 2 Department of Mathematics and Statistics, San Diego State University, San Diego, CA 92182-7720, USA; psalamon@sdsu.edu

## 1. Background

The year 1975 marked the beginning of an entirely new direction for thermodynamics with the publication of Curzon and Ahlborn’s innocent-looking short paper “*Efficiency of a Carnot engine at maximum power output*” [1]. That paper not only ignited what became known as Finite-Time Thermodynamics (FTT), it is also an important story about how significant new ideas come out of casual conversations, totally unplanned, and all the trouble the originators have to go through to get their ideas published. We are very fortunate that Boye Ahlborn and Frank Curzon were willing to tell us about the birth and publication troubles of the main idea behind FTT in their paper “*The hunt for new understanding*” in this volume. The fact that overcoming the obstacles ended in success should be an encouragement to all young scientists with unconventional ideas: Persevere and you will win.

One thing is writing the “spark” that starts a new development. But that only works if the scientific environment is ready for the change. In 1975, three additional facts came together. (1) Prof. R. Stephen Berry had recently taken up his position at the University of Chicago. The terrible air quality in Chicago at the time reminded him of his earlier interest in incorporating time into thermodynamic analyses and prompted him to start pondering possible ways of reducing waste in industrial processes. (2) Prof. Peter Salamon had just started his Ph.D. studies under Prof. Berry with a desire to work out a differential geometrical formulation of thermodynamics. Finally, (3) Prof. Bjarne Andresen had just come to postdoc with Prof. Berry on atomic collision theory. This small diverse group caught the generality and importance of Curzon and Ahlborn’s result restricting the duration of a thermodynamic process. Time is fully as important as a thermodynamic variable as the various free energies are, not merely as an evolution parameter used in kinetics, but as a key ingredient in calculating realistic efficiencies. For the full details, see [2]. The scientific world was ready for this new view on thermodynamic optimization, and many young researchers soon joined the effort.

To be honest, the idea of limiting the duration of a thermodynamic cycle had appeared in print earlier than the Curzon–Ahlborn paper, but for less general systems, in conference proceedings, and in local languages (see [3] for a historical account). All of these circumstances prevented the idea from being noticed. It only caught the attention of the scientific community as presented in 1975 by Curzon and Ahlborn, unaware of the previous attempts.

Our own first paper on FTT from 1977 [4] was the first to pick up the idea and coin its name; that is why we celebrate the 50th anniversary of Finite-Time Thermodynamics this year. Actually, we were in a dilemma as to whether the celebration should include a Special Issue of *Entropy*, considering that we, for unrelated reasons, collated an FTT Special Issue just a few years ago [5]. In the end, we decided that the importance of the 1975 paper was large enough to carry another Special Issue. We hope that you agree.

Some people have argued that Finite-Time Thermodynamics was not a good name for what the topic has evolved into, e.g., that it should have been called Finite-Resource Thermodynamics instead. To a certain extent, we understand such wishes since many of the ideas behind FTT have turned out to be so general that they also apply to limitations on other quantities like heat exchange area, chemical reaction rates, memory, and capital. Furthermore, the basic theory to be extended does not have to be thermodynamics, it can also be economics, computer science, or other disciplines. Thus, “time” in FTT can be read in a very general way as anything, which is limited in a dynamic system by construction.

However, for thermodynamics, time is a very special quantity. Besides designating the direction of evolution, as it does also in many other fields, it shares with extensive variables the property that it measures one of the net changes in a process. The traditional naming of thermodynamics was always a little inappropriate since the dynamics are not specified and in fact it was traditionally what has come to be called comparative statics—enabling the comparison of the static initial and final equilibrium states. The net changes in the extensive variables traditionally gave bounds on the possible values of the process variables such as work, heat, and entropy production associated with the process. Our original goal in extending thermodynamics to finite time was to add one more net effect: the time elapsed. This additional net effect would result in more stringent limits on the process variables while keeping the comparative statics framework intact, i.e., keeping the limits dependent only on the net effects of the process. This comparative statics framework had previously been borrowed from thermodynamics for economic analyses [6] where it is oft used to this day.

The first focus of FTT was the limited time allotted to the completion of the chosen process and the influence that this has on the efficiency of that process. A second important element in FTT is the definition of a metric among the relevant thermodynamic variables from which a length of the reaction path can be calculated and in turn used to bound the dissipation in that process. Such a metric was defined in 1980 [7], curiously growing out of a re-formulation of the Maxwell relations in quantum–mechanical bra–ket notation [8], in an effort to make them more understandable. This metric, available in a wide selection of basic coordinates, only further expanded the generality of FTT, e.g., into statistical mechanics, information theory, and quantum mechanics.

## 2. Content

We start out this Special Issue celebrating the first 50 years of Finite-Time Thermodynamics with Boye Ahlborn and Frank Curzon’s own account of how they got the idea and the trouble they had getting it published, “*Maximum Power Efficiency*”.

Next, Anatoly Tsirlin, Alexander Balunov, and Ivan Sukin give a review on how the ideas of FTT are equally useful within economic theory in “*Finite-time thermodynamics: problems, approaches, results*”.

Gregory Behrendt and Sebastian Deffner go beyond the usual chemical energy range and analyze “*Endoreversible Stirling cycles: plasma engines at maximal power*”, showing that only the functional form of the equation of state being linear in temperature and additive in volume are important, not its further details.

Also Xiu-Hua Zhao and Yu-Han Ma go back to basics and provide a closer look at the historical Yvon engines in “*Revisiting Endo-Reversible Carnot Engine: Extending the Yvon Engine*”.

In a more practical approach, François Lanzetta focuses on “*Compressor power and efficiency optimisation: a finite time thermodynamics approach*”. In particular, he studies the influence of suction and discharge tube diameters and of gas pressures.

Stability of finite-time engines against perturbations of their assumed path is important. Julian Gonzalez-Ayala, David Pérez-Gallego, Alejandro Medina, José M. Mateos Roco, Antonio Calvo Hernandez, Santiago Velasco, and Fernando Angulo-Brown analyze the stability regions for different optimality criteria in their paper “*Linking optimization success and stability of finite-time thermodynamics heat engines*”.

In “*Re-Evaluation of the Extremum Rate of Entropy Production Principles and the Fourth Law of Thermodynamics*”, Anil Bhalekar and Vijay Tangde attack the issue of stability in more general terms by using Lyapunov stability theory to connect the Fourth Law of thermodynamics to the rate of entropy production and how to control it.

Related to this, Sergey Amelkin shows that any macrosystem, thermodynamic or not, described by extensive (i.e., proportional to size) variables and under the influence of intensive (i.e., independent of size) forces may be described by the same theory; “*Thermodynamic Theory of Macrosystems: Entropy Production as a Metric*”.

Ricardo T. Páez-Hernández, Juan Carlos Pacheco-Paez, Juan Carlos Chimal-Eguía, Delfino Ladino-Luna, and Javier Contreras Sánchez describe the efficiency of their heat exchangers in terms of their respective ”Number of Transfer Units” (NTUs) and distinguish between a number of performance situations using different objective functions in “*Transfer irreversibilities in the Lenoir cycle: FTT design criteria with e-NTU*”.

Since his initial papers mentioned above [8], Frank Weinhold has used his bra–ket notation and associated reaction coordinates widely in atomic physics. In “*Thermodynamics of Intrinsic Reaction Coordinate (IRC) Chemical Reaction Pathways*” he describes many uses of such state-dependent intrinsic reaction coordinates focusing on the full reaction paths, not just the initial and final states.

Abhishek Dutta, Bitan Mukherjee, Sk Aftab Hosen, Meltem Turan, Denis Constales, and Gregory Yablonsky solve chemical kinetics rate equations using artificial intelligence in the form of so-called “physics-informed neural networks” (PINNs), i.e., the physical and chemical equations are built into their training data sets. The results for “conservatively perturbed equilibrium” systems are presented in “*A Physics-informed neural network (PINN) approach to over-equilibrium dynamics in conservatively perturbed linear equilibrium systems*”.

In “*Finite-Time Thermodynamics and Complex Energy Landscapes: A Perspective*”, Christian Schön has written an extensive review on how FTT is being used successfully to generate efficient pathways for the synthesis of desired molecules.

Finally, Bernard Guy explores the philosophical aspects of FTT by connecting it to relativity for a geological example in “*(Finite-Time) Thermodynamics, Hyperbolicity, Lorentz Invariance: Study of an Example*”.

## 3. Generality of FTT Ideas

Thermodynamics, originally developed by Carnot to model the efficiency of heat engines, was soon after extended by Gibbs to analyze chemical reactions. Similar extended analyses have had unbelievable applicability in other areas as well, e.g., computer science, quantum mechanics, and economics. The reason is that thermodynamics is really a theory (description) of the flow of conserved quantities, driven by a relevant force, through surroundings that resist such flow. Many natural as well as abstract setups fit this general description. Hence, it is not surprising that thermodynamic ideas may be carried over to seemingly unrelated disciplines ranging from real applications in engineering to abstract information handling in computer science, economic trading, and beyond. That, of course, goes for FTT as well. This feature has also made it a lot of fun to work in this area, offering chances to interact with colleagues from many different specialties.

What ties all the many uses of FTT together is “the cost of haste”. Quite universally, as soon as you try to push any dynamic system to go faster, you need to increase the gradients driving it. FTT tells you how to do it wisely, i.e., with a minimum of dissipation. Importantly, that is not necessarily the path that free-running kinetics would take. Rather, the path generally should be uniformly close to the reversible (infinitely slow) path. The driving sequence necessary to achieve the optimal path is sometimes called counter diabatic [9]. The most recent development in this direction includes the cost of measuring (checking) the actual time evolution of that path. This involves a balance between energy, speed, and accuracy [10], a very interesting combination.

## 4. Where Is FTT Headed?

It is hard to imagine any field where the finite-time concept will not have an important role. Humans are impatient, so anything we want to do, be it in science, technology, economics, or psychology, should be completed as quickly as possible and at as low a cost as possible, i.e., minimizing “the cost of haste”. At the end of our previous Special Issue [11], we concluded with a summary entitled “Future perspectives of Finite-Time Thermodynamics” in which we were quite specific about several future avenues.

The one additional specific thermodynamic development we would hope to see in the near future is a theory for counting *compensated dissipation* in analogy to Clauisus’ concept of “uncompensated heat” [12] as the work not harvested in a process but dissipated as heat. With such a measure we could examine real processes and have some clear methods for counting how much of the dissipation in the process is inescapably due to each of the different constraints acting in the process. Methods for accounting losses in this manner would be invaluable for process design and for understanding entropy production in biological processes. In this latter direction, we would hope for methods to spell out the entropy production that must be present to account for the choreography involved in the various steps of a process and in the material translocation of the reactants and products for the various steps of a complex reaction network.

Many things have been developing as we forecasted back then, but one direction has gone faster than our prior expectation: AI (artificial intelligence). Being a massively large-scale informational process, it naturally also follows thermodynamic/statistical mechanical principles and its workings can be optimized for efficiency. Not least when going to the next level, AGI (artificial general intelligence), the efficiency in time, power consumption, and accuracy of results will continue to be very important.

Other fertile directions also remain. In particular, we expect further breakthroughs based on counter diabatic driving, quantum computing, and in pushing our understanding further along biochemical pathways.

## 5. Lessons Particularly for Young Researchers

- Believe in what you are doing regardless of opposition from the establishment.
- Look for concepts and principles and search for equivalents outside your main field. Your results will undoubtedly apply there too, under different names. Have an open mind, but be quantitative.
- You did not work hard in research just to solve somebody else’s problem, but to search for insight into unknown territory.
